# Supplementary material for: Maximizing regional biodiversity requires a mosaic of protection levels
Source: PLoS Biol. 2021 May 19;19(5):e3001195. doi: 10.1371/journal.pbio.3001195 (PMC8133472; doi:10.1371/journal.pbio.3001195)
Supplement: S1 Table — (PDF) [file pbio.3001195.s001.pdf]

		Number of Protected areas	Number of Surveys/Transects/Roads
Birds	SPA	132	133 (50 stops by roads)
	RA	283	486 (50 stops by roads)
	NPA		1792 (50 stops by roads)
Reef-fishes	SPA	25	1306
	RA	19	504
	NPA		346
Plants	SPA	5	3064
	RA	191	32253
	NPA		6973
