# Supplementary material for: Maximizing regional biodiversity requires a mosaic of protection levels
Source: PLoS Biol. 2021 May 19;19(5):e3001195. doi: 10.1371/journal.pbio.3001195 (PMC8133472; doi:10.1371/journal.pbio.3001195)
Supplement: S2 Table — (PDF) [file pbio.3001195.s002.pdf]

DATA	SOURCE	LINK
**Data**		
Reef fish data	RLS: Edgar GJ, Stuart-Smith RD. Systematic global assessment of reef fish communities by the Reef Life Survey program. Scientific Data. 2014;1:140007<br><br>Cinner JE, Huchery C, MacNeil MA, Graham NA, McClanahan TR, Maina J, et al. Bright spots among the world's coral reefs. Nature. 2016;535(7612):416.	https://reeflifesurvey.com/reef-life-survey/survey-data/<br>https://www.nature.com/articles/nature18607
Bird data	BBS	https://www.pwrc.usgs.gov/bbs/.
Alpine plant data	Thuiller W, Guéguen M, Georges D, Bonet R, Chalmandrier L, Garraud L, et al. Are different facets of plant diversity well protected against climate and land cover changes? A test study in the French Alps. Ecography. 2014;37(12):1254-66	http://www.cbn-alpin.fr/
Official coverages of protected areas	U.S. Geological Survey<br><br>WDPA	https://gapanalysis.usgs.gov/padus/<br><br>https://www.protectedplanet.net/.
Land cover in US	U.S. Geological Survey	https://www.sciencebase.gov/catalog/item/5a1c35ade4b09fc93dd63fc4
Land cover in France	THEIA-LAND	http://www.theia-land.fr/.
