# Supplementary figures and images for: Maximizing regional biodiversity requires a mosaic of protection levels
Source: PLoS Biol. 2021 May 19;19(5):e3001195. doi: 10.1371/journal.pbio.3001195 (PMC8133472; doi:10.1371/journal.pbio.3001195)

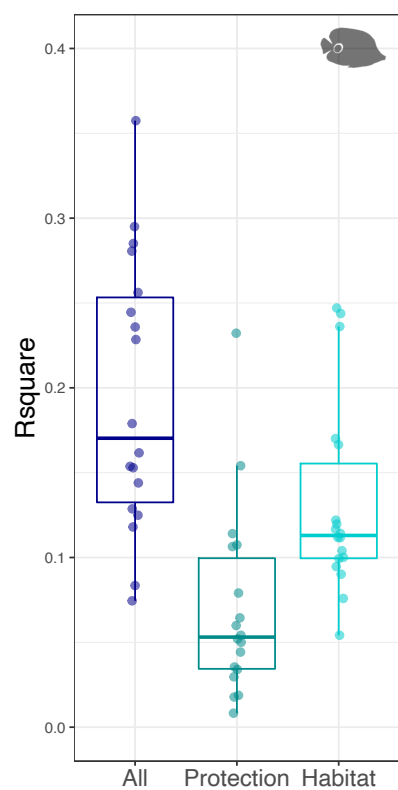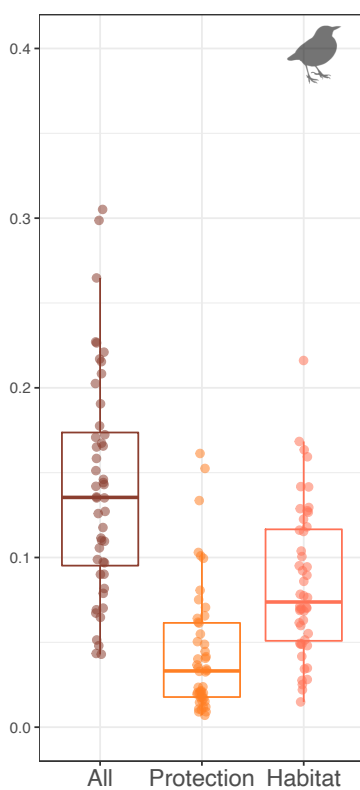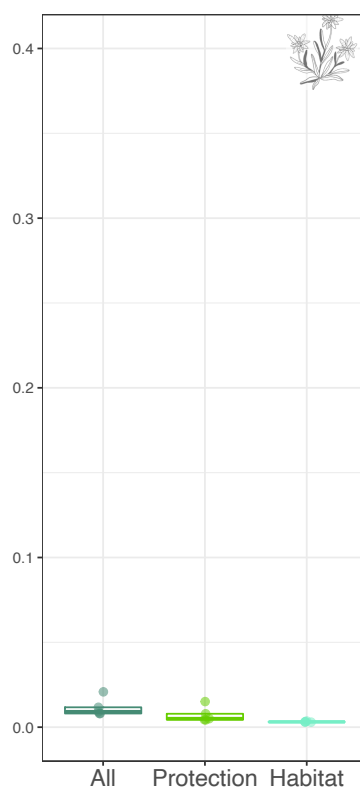

Supplement: S1 Fig — Rsquare of the marginal effect of protection and habitat to observed turnover between Strictly Protected areas surveys and surrounding surveys within a 50-km buffer (from non-protected areas and/or restricted areas (n = 18, 131, 5) for reef fishes, birds, and alpine plants. Some Strictly Protected was removed because habitat was strictly identical between surveys making impossible comparison of the relative contribution of both protection and habitat. The raw data can be found in https://github.com/LoiseauN/Betadiversity-protected-areas. (PDF) [file pbio.3001195.s003.pdf]

100 km

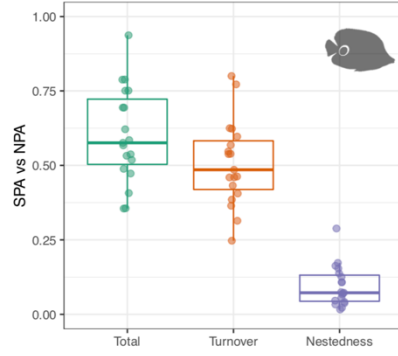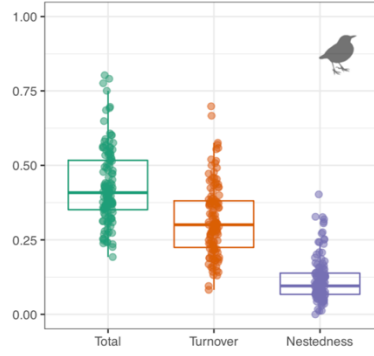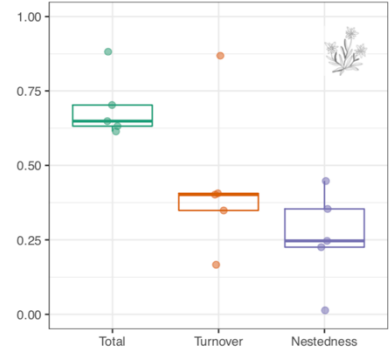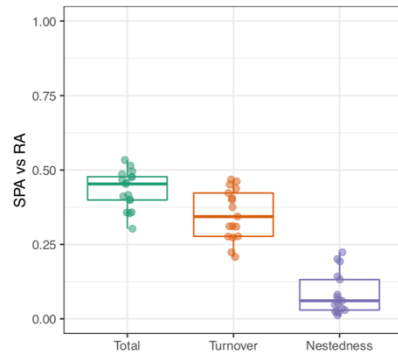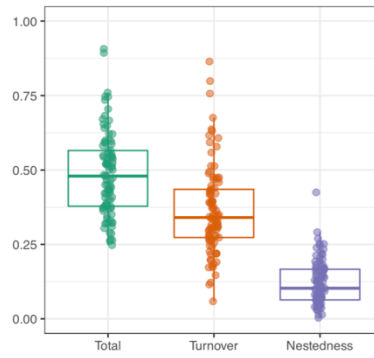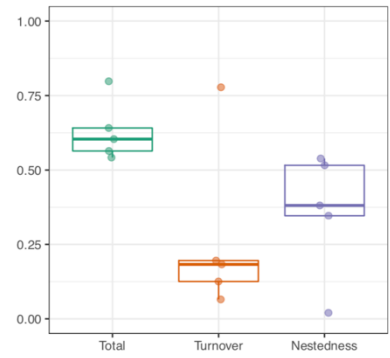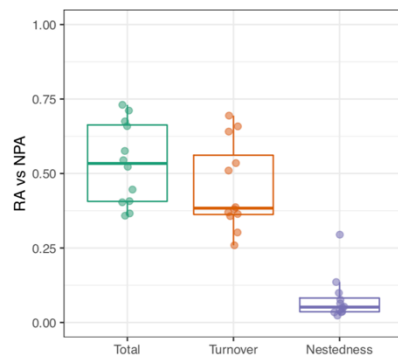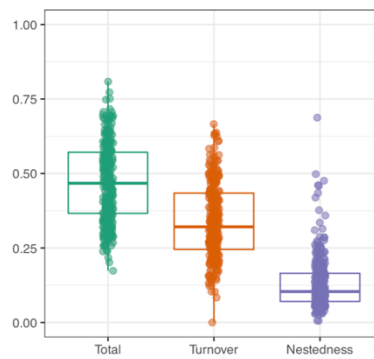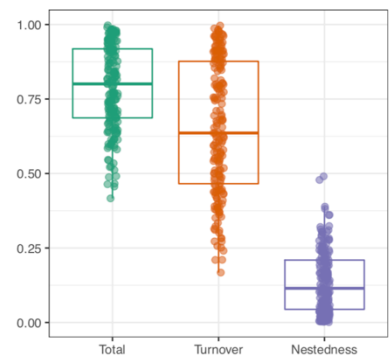

10 km

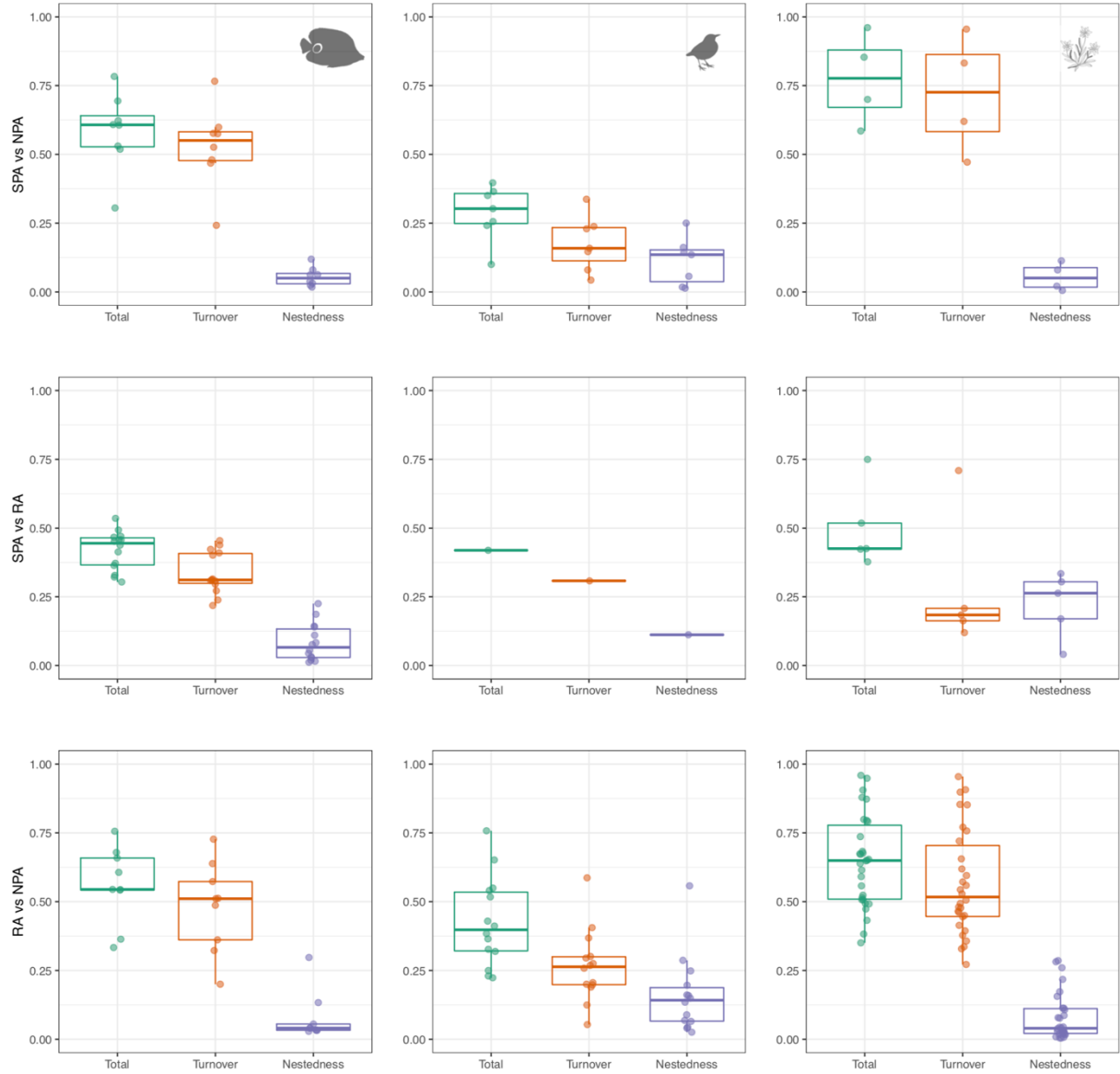

Supplement: S3 Fig — The raw data can be found in https://github.com/LoiseauN/Betadiversity-protected-areas. NPA, Non-Protected Area; RA, Restricted Area; SPA, Strict Protected Area. (PDF) [file pbio.3001195.s005.pdf]

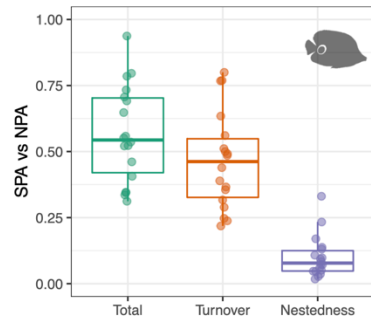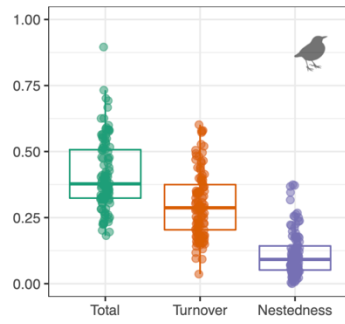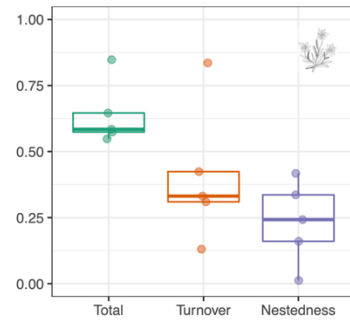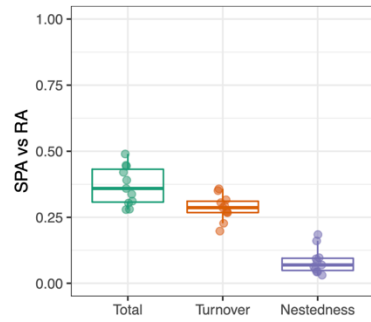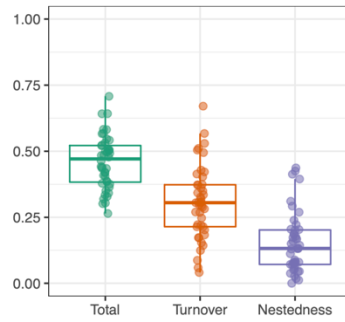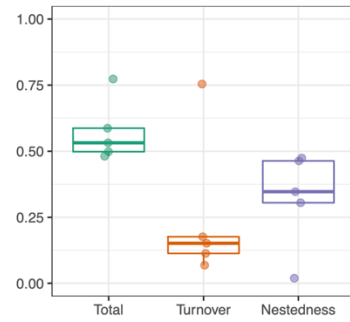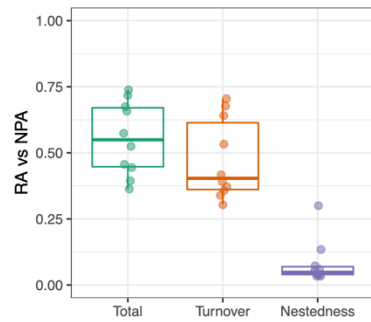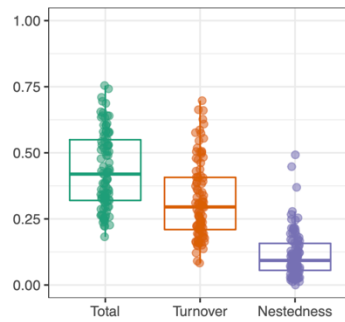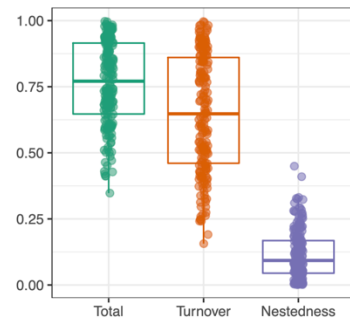

Supplement: S4 Fig — The raw data can be found in https://github.com/LoiseauN/Betadiversity-protected-areas. NPA, Non-Protected Area; RA, Restricted Area; SPA, Strict Protected Area. (PDF) [file pbio.3001195.s006.pdf]

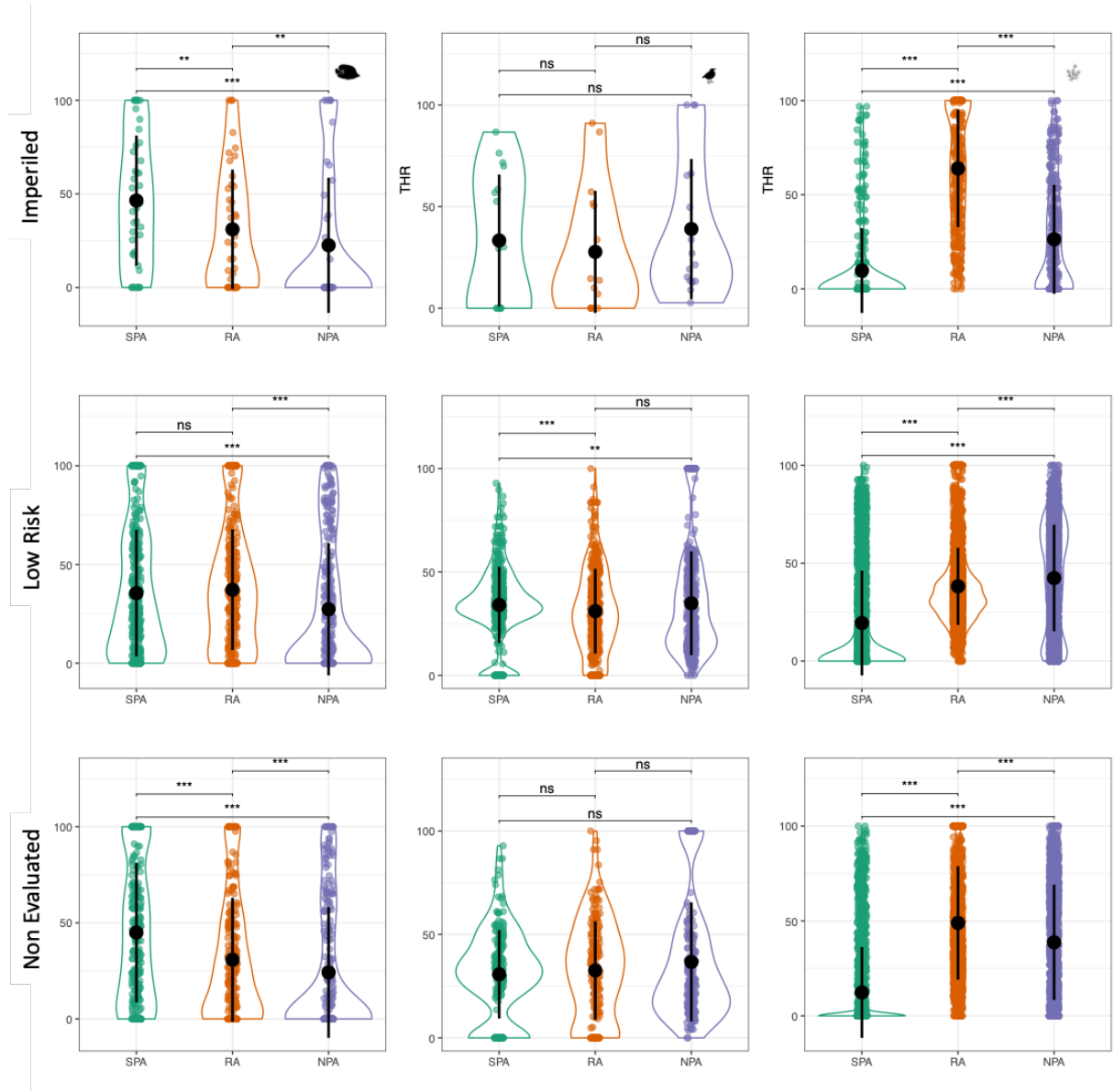

Supplement: S5 Fig — CR, EN, VU, and NT species as “Imperiled”; LC as “Low risk.” Stars indicate significance of Tukey post hoc test computed after the analysis of variance. *** p-value < 0.01, ** p-value < 0.05, * p-value < 0.1. The raw data can be found in https://github.com/LoiseauN/Betadiversity-protected-areas. CR, Critically Endangered; EN, Endangered; LC, Least Concern; NPA, Non-Protected Areas; NT, Near Threatened; RA, Restricted Areas; SPA, Strictly Protected Areas; VU, Vulnerable. (PDF) [file pbio.3001195.s007.pdf]
