# Supplementary material for: Maximizing regional biodiversity requires a mosaic of protection levels
Source: PLoS Biol. 2021 May 19;19(5):e3001195. doi: 10.1371/journal.pbio.3001195 (PMC8133472; doi:10.1371/journal.pbio.3001195)
Supplement: S2 Fig — Bold black dotted line indicate 0 SES, for random values. Black dotted lines indicate the α = 0.05 threshold of 1.96 SES for significantly nonrandom values. Each data point represents a comparison between protected area and its proximate less protected area (in a buffer of 50 km, 100 km, and 10 km). The raw data can be found in https://github.com/LoiseauN/Betadiversity-protected-areas. NPA, Non-Protected Area; RA, Restricted Area; SES, standardized effect size; SPA, Strict Protected Area. (PDF) [file pbio.3001195.s004.pdf]

Null models

We tested whether our observed *ß*-diversity values among levels of protection were larger or smaller than expected under a random assembly model. For each protected area and for each iteration of the bootstrap, we generated 1000 random species assemblages using the curveball algorithm. This algorithm maintains species frequency and sample species richness while shuffling species co-occurrences across cells.

The large majority of SES values are positive and significantly higher than expected by chance for total species dissimilarity and turnover (Fig. 3) suggesting that the differences in species composition between levels of protection are not simply due to a random distribution of species among sites. However, turnover in reef fish species between restricted areas and non-protected areas is not significantly different than expected by chance highlighting that only strictly protected areas play a key and unique role in sustaining regional fish biodiversity (Fig. 3, G).

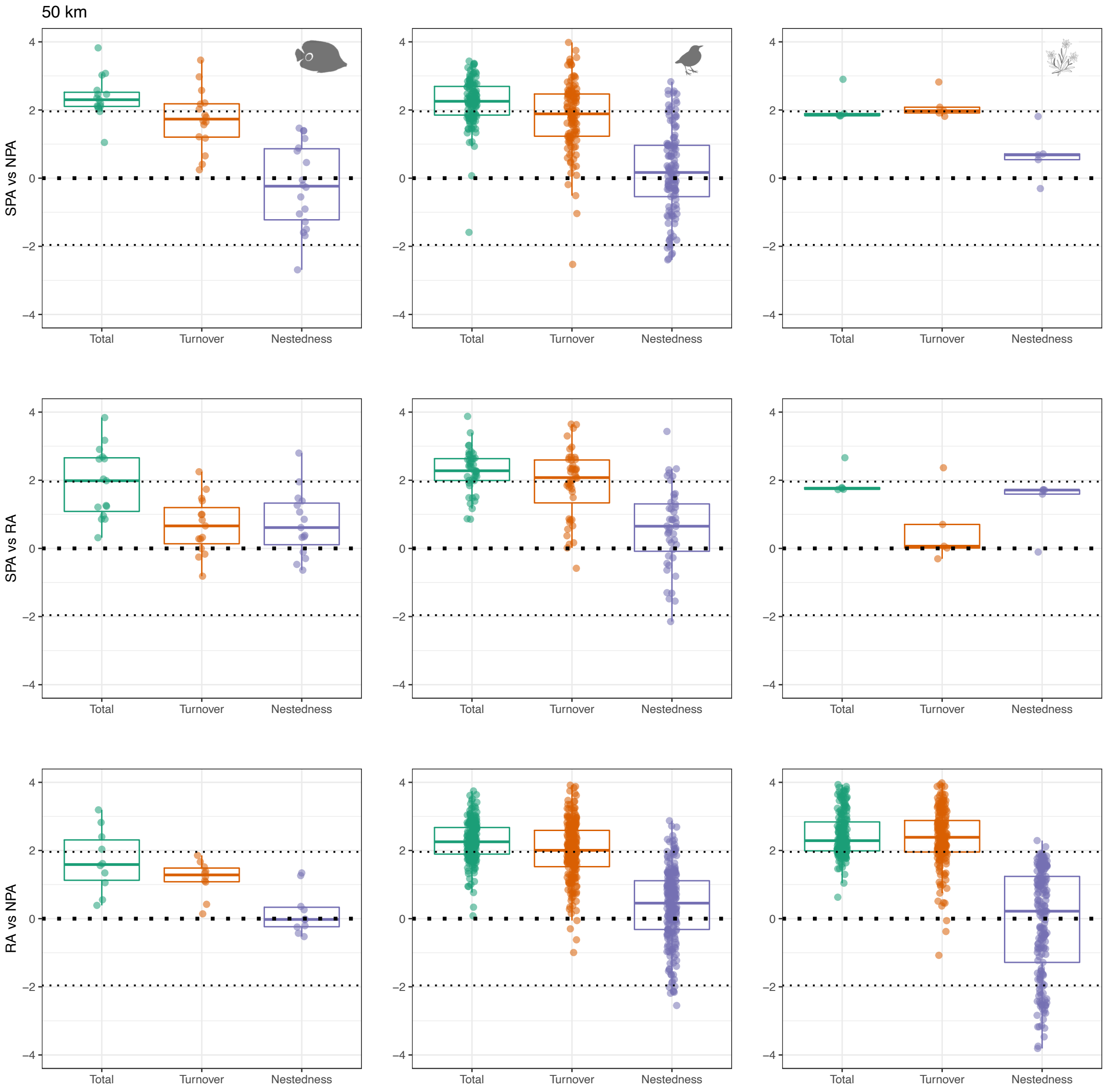

50 km
SPA vs NPA
SPA vs RA
RA vs NPA
4
2
0
−2
−4
Total
Turnover
Nestedness

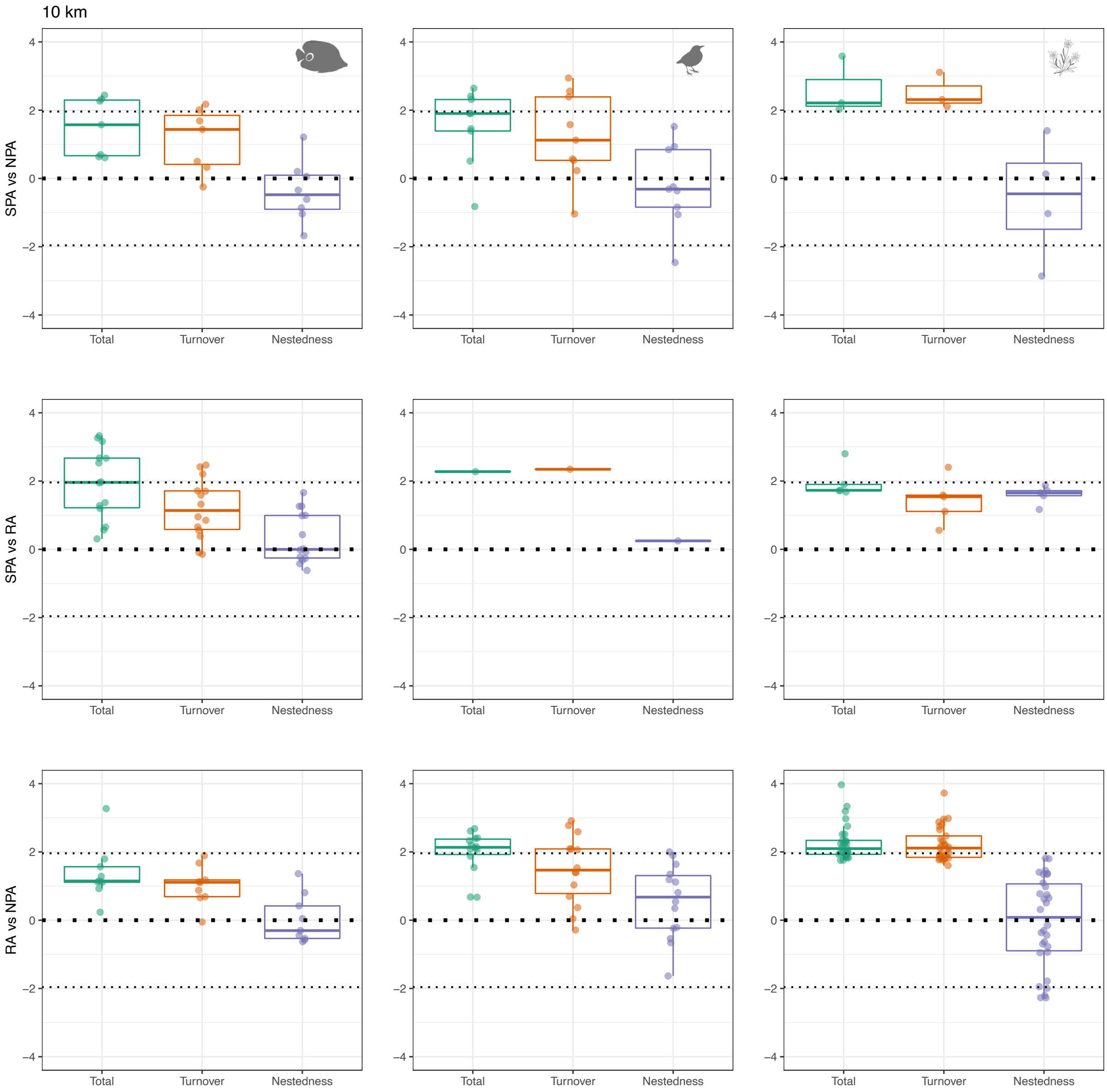
10 km
SPA vs NPA
SPA vs RA
RA vs NPA
4
2
0
−2
−4
Total
Turnover
Nestedness

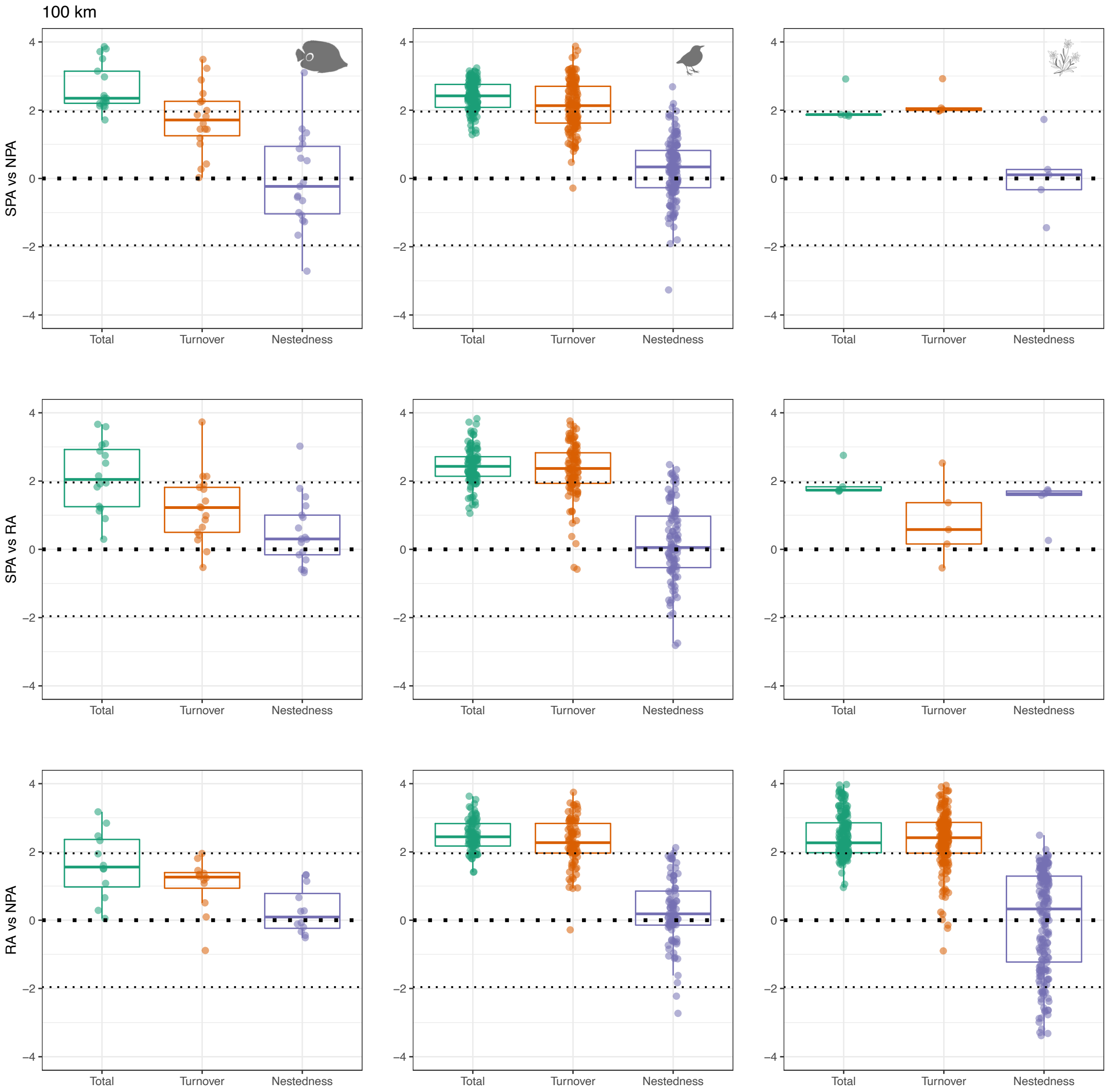

100 km
SPA vs NPA
SPA vs RA
RA vs NPA
Total
Turnover
Nestedness
4
2
0
−2
−4
