# Supplementary material for: Maximizing regional biodiversity requires a mosaic of protection levels
Source: PLoS Biol. 2021 May 19;19(5):e3001195. doi: 10.1371/journal.pbio.3001195 (PMC8133472; doi:10.1371/journal.pbio.3001195)
Supplement: Attachment — Submitted filename: responsetoreviewers.docx [file pbio.3001195.s008.docx]

Previous interaction: [PBIOLOGY-D-20-01943R1]

Dear Editor,

We would like to thank you for allowing us to resubmit our manuscript entitled “Maximising regional biodiversity requires a diversity of protection levels” PBIOLOGY-D-20-01943R1 for publication in *Plos Biology*. We are also grateful to the associate editor and the reviewers for their useful comments which significantly improved our manuscript. We hope that this revised version addresses their concerns.

The main reviewers’ criticism was about environmental and habitat conditions that can blur our results about protection effects on biodiversity. In brief, for each protected area, we applied a set of two distinct but interrelated statistical analyses at two spatial scales to fully assess the influence of both protected areas and environmental conditions. Indeed, we coupled our previous approach linking species turnover to habitat dissimilarity, with a second approach, a distance-based redundancy analysis (dbRDA), to analyze changes in species assemblages among samples in relation to the protection level and habitat. This analysis reinforces our main finding that differences in habitat cannot explain, alone, the high species turnover between different protections levels. Last but not least, null models already used in the previous manuscript provide a robust test of the protection influence regardless of rarity, species occurrences and habitat. All together these analyses show that our results on the influence of protection on species composition across three taxa and 655 protected areas are holding tight.

Finally, even if our results suggest that species turnover is partly dependent on habitat, the consequence in terms of regional biodiversity conservation is still valid with Strictly Protected, Restricted and Non-Protected areas not sharing a non-negligible part of the species regional pool so being complementary.

As mentioned by reviewers, this result is totally new, timely and important with deep implications on biodiversity management at the regional scale when a broad coalition of organizations advocate to protect 30% of land and sea by 2030. We also expanded our discussion to better mention the influence of potential confounding environmental factors on protection effects.

Thank you sincerely for your time and effort in handling and reviewing our manuscript. The three reviewers provided important criticisms that greatly improved the quality of this manuscript and encouraged the authors to think critically about the results while recognizing its breakthrough message for three major taxa under threats.

Below we present point-by-point responses to the concerns raised by the reviewers. (Important changes according to concerns of reviewers are highlight by red marks in the main manuscript)

We hope this revised version fulfils your expectations and meets your requirements.

Sincerely,

On behalf of all coauthors

Nicolas Loiseau

Dear Dr Loiseau,

Thank you very much for submitting your manuscript entitled "Maximising regional biodiversity requires a diversity of protection levels" for review as a Research Article by PLOS Biology. As with all papers reviewed by the journal, yours was assessed and discussed by the PLOS Biology editors, by an academic editor with relevant expertise and by three independent reviewers (the Academic Editor also added a few technical concerns - see the foot of this email). Please accept my apologies for the unusual time that it's taken for us to secure reviews over this particularly challenging period.

Based on the reviews, I regret that we will not be able to accept this manuscript for publication in the journal. I hope that you will find the reviewers' feedback helpful as you consider how to proceed with this work.

The rationale behind this decision is that the comments of reviewers #1 and #2 will need a substantial amount of effort to address. In particular, the Academic Editor thought that their two main comments about the potential for environmental conditions to explain the study results and representativeness of the sampled habitats were absolutely fundamental to this story holding tight. The Academic Editor asked me to stress that you will need to address these points in a convincing and robust way through additional analyses, in order to achieve the level needed for PLOS Biology.

At this point, we can't know whether new analyses would support your conclusions, and as generating these would require significant effort, we cannot in good faith invite resubmission. However, if you are willing to take on the extra work involved and comprehensively address the reviewers' concerns - and I must emphasize that this would be your choice - we would be willing to consider an extensively revised version as a new submission. As such, the article would receive a new number and submission date and we would evaluate it against any work published in the interim that might undermine the novelty. Although we would try our best to engage the same academic editor and reviewers, please be aware that this isn't always possible. Also, if the new experiments and data introduce new areas that require review we might need to consult additional/new reviewers. The new data would all need to be evaluated as if a first submission and further revisions might prove necessary.

We appreciate that the scale of the requested additional work is significant and you may well prefer to pursue publication of this work elsewhere. At this point, your manuscript is no longer under active consideration at PLOS Biology.

Please do feel free to contact me if you would like to discuss next steps for your manuscript. We might also be able to facilitate a faster consideration at one of the other PLOS journals; if this is of interest to you, please let me know.

If you do make the choice to revise and re-submit to PLOS Biology, you should provide a point-by-point response to the reviewers' comments when re-submitting. Please include this as a separate file, labelled 'Response to Reviewers'. Please also provide the tracking number of this manuscript [PBIOLOGY-D-20-01943R1] in the 'previous interaction' section of the online submission form and in your cover letter when re-submitting.

Sincerely,

Roli Roberts


Roland G Roberts, PhD,
Senior Editor,
[rroberts@plos.org](mailto:rroberts@plos.org),
PLOS Biology

------------------------------------------------------------------------
REVIEWERS' COMMENTS:


Reviewer #1:

This study uses several datasets (fish, birds, plants) from different management types to explore how habitat protection influences local and regional diversity, via influence on aspects of beta-diversity. The overarching idea is that while management levels don't seem to be influence local (alpha) diversity much, they are influencing the nestedness/turnover aspects of species composition. Thus, species appear to be 'sorting' among different management levels, and the full complement is needed to maintain larger scale diversity.

In principle, I think the overall question is interesting and useful. It's also pretty well known by now that species richness tells us nothing about species composition and that different types of species can tolerate different types of habitats (including different types of management). So, my first question is what the novelty of the study is. It's nice to explore it 'big' with really great datasets, but I'm not sure we learn a lot new here relative to analyses that have been done.

**[R1.1]** We agree that it is well known that species richness tells us nothing about species composition and turnover across scales. We believe that the novelty of our paper lies in the strong species turnover between adjacent protected and not protected areas that cannot be solely explained by habitat and environmental differences. Our results shed light on how protection can shape biodiversity patterns at the regional scale across three taxa. This has never been addressed before and has important implications when a broad coalition of organizations advocate to protect 30% of land and sea by 2030. See line 49

Furthermore, it would seem that some exploration is needed as to why results here, with little influence on alpha diversity, differ from e.g., results from the PREDICTS database (where reductions in alpha diversity were common). I guess this is probably an issue of 'scale' and intensity of human encroachment.

**[R1.2]** This statement certainly refers to the paper entitled *Local biodiversity is higher inside than outside terrestrial protected areas worldwide* (<https://www.nature.com/articles/ncomms12306>) showing that globally, “species richness is 10.6% higher inside protected areas compared to the outside”.

This difference from our results can be due to several factors:

First, this major article combines 13,669 species of vertebrates, invertebrates and plants while we analyzed taxa separately.

Second, we used geographical buffers around protected areas, allowing to control the spatial extent of comparisons that are made. We only assess species differences between neighbor areas. By contrast, the PREDICTS database defines sites to be in the same study if they were sampled using the same methodology and the data were reported in the same paper, thus the spatial extent of pairwise comparisons (outside vs inside) is variable. That being said we thank the referee for this suggestion and now discuss this difference see line 249

*This contrasts with some studies showing that species richness is 10.6% higher inside protected areas compared to the outside{Gray, 2016 #227}. However, this difference may be due to the difference in spatial extent of pairwise area comparisons and species analyzed between studies since our approach is based on within regional biodiversity patterns using null models on three different taxa.*

Reference:

Gray, Claudia L., et al. "Local biodiversity is higher inside than outside terrestrial protected areas worldwide." *Nature Communications* 7.1 (2016): 1-7.

Newbold, Tim, et al. "Global effects of land use on local terrestrial biodiversity." *Nature* 520.7545 (2015): 45-50.

While the main question in the manuscript was on beta-diversity, I was rather disappointed with the rather simplistic analysis comparing habitat differences and protection status.  There are very useful and sophisticated tools available in the literature (e.g., multivariate partitioning, jSDMs, etc.) that are well suited to separate the effects of habitat differences and its interaction with protection level. The way the analyses were done here seem preliminary at best.  With the data available on habitat and protection level, I think this would need to be the primary analysis, not a secondary analysis 'afterthought'.  Clearly, there is always distance decay effects on beta diversity—these result from difference in environment and spatial factors. The question then is how does 'protection' interact with the general background variation in beta diversity. Again, the analyses done are heading in this direction, but as variation in beta-diversity is the main question of interest here, I would have expected a bit more depth on the analysis and the partitioning of effects. The null model gets at some of this, but not the full picture.

**[R1.3]** The three reviewers pointed out this issue, so more clearly and quantitatively disentangling the effects of protection and habitat. We have now re-worked extensively on data and new analyses to address this important issue.

1. We provided a more complete description of environmental data and associated analyses.
2. As suggested, we used a distance-based redundancy analysis (dbRDA) to analyze changes in species composition between samples in relation to the protection level and two habitat PCA axes. Significance of the models as well as the significance of each axis and of the marginal effects were tested using ANOVA-like permutation tests. We then used a partial dbRDA, using the Jaccard turnover component, to test the effect of protection after accounting for habitat and vice-versa.
3. We also show that the habitat issue is circumvented by only considering less-strictly-protected areas within a 50-km buffer of the more protected areas (with similar results at 10 and 100km). Changes in environmental conditions should thus remain relatively limited over such spatial scales.
4. For plants, where the influence of habitat strongly explains species turnover between non-protected and protected areas, we chose a small buffer (1 km) as a reasonable range at which habitats can shape plant species occurrences. This choice is highly conservative since the probability to have different habitats between samples is higher than at larger buffer size which may cover several habitats.
5. We would like to remind that the null models we used are also a strong way to test the influence of protection on species composition while controlling for rarity, species occurrence patterns and habitat.

Altogether, we believe our results support the major role of protection in shaping species assemblages at the regional scale. Line 94

*At the scale of individual surveys, we observe a high species turnover within regions with on average 0.62 (+/- 0.14) for fish, 0.64 (+/- 0.17) for birds, and 0.97 (+/- 0.01) for plants. The distance-based redundancy analyses (dbRDA) reveal that both habitat and protection significantly explain this turnover in species composition between surveys of the same region (overall F-test = 2.8±1.9, 9.3±5.7, 35.4±9.7, mean adjusted R2 = 0.21±0.1, 0.14±0.06, 0.01±0.05, for fishes, birds and plants, respectively Fig. S1 Appendix). Yet, explanatory power of dbRDA remains low (mainly due to the sampling effect), particularly for plants, suggesting that, at the scale of surveys, many other micro-habitat variations may drive species composition beyond protection and macro-habitat effects.*

*Partial dbRDA reveal that habitat, after controlling for protection level, significantly explains species turnover between surveys of the same region for the three taxa (Fig. 2). Symmetrically, protection, after accounting for habitat, also significantly explains species turnover for the three taxa (Fig. 2). On the balance, habitat has more influence than protection on species turnover between surveys of the same region for birds (70% vs. 30%) and fish (69% vs. 31%) but this is the opposite for plants (28% vs. 56%). These results highlight that differences in habitat cannot explain, alone, the high species turnover between surveys of the same region. Since most of variation in species turnover remains unexplained between surveys within a region, owning to sampling “noise” and unmeasured micro-habitat variations, we then pool surveys by protection level for a given region with the same sampling effort in each protection level (see Methods) for the following analyses.*

I also felt that the entire discussion of beta diversity was superficial and missed what may seem subtle, but are actually critical differences. In particular, the authors discuss the question of alpha, beta, gamma diversity as a diversity partition via Whittaker or related.  However, the actual beta diversity measured used was Jaccard's similarity. This is in fact, what most authors do, and in the limit when the number of sites is 2, pairwise metrics like Jaccard's and beta diversity are the same.  However, pairwise measures of beta diversity can really diverge from Whittaker's beta diversity when there are multiple sites in a region.

**[R1.4]** We fully agree with this remark, we now discuss carefully beta diversity. We also added information in the Methods on the way we computed beta diversity.

Here, the authors talk about gamma diversity multiple times, but never actually measure it because their measure of beta diversity can be quite different from whittaker's beta.  I would suggest that if the author really want to think about questions like 'homogenization', which they discuss frequently, they actually have to really measure gamma diversity. Homogenization can differ when comparing pairwise and total beta diversity. Furthermore, the discussion of homogenization itself is much more nuanced than implied by the authors, and I would suggest they take a look at e.g., Socolar et al 2016 in TREE to see a bit more about how human activities can both increase or decrease similarity and beta-diversity. Only by actually measuring real gamma and partitioning the possible influences on it, can we start to understand how species compositional differences are influenced by management and other factors.

**[R1.5]** We disagree with the referee on this last point. The exact partition of beta diversity is ideal for revealing the spatial scaling of diversity loss. However, this classical approach cannot disentangle the nestedness and turnover components (Baselga 2010). High species turnover among sites would imply that conservation must target multiple sites, while nestedness suggests that conservation might target the richest sites. Socolar et al. (2016) highlighted the value of nestedness/turnover approach: *“Turnover across natural sites implies that conservation must target multiple sites, while nestedness suggests that conservation might target the richest sites.”*

In the same vein, the Jaccard index is widely used in studies on biotic homogenization (Legendre and Legendre, 1998, McKinney and Lockwood, 2001 and references therein)

That being said we strongly limited our discussion about homogenization, that we do not fully address since it may require species assemblage dynamics through time.

Reference:

Baselga, A. (2010). Partitioning the turnover and nestedness components of beta diversity. Global ecology and biogeography, 19(1), 134-143.

Kühn, I., & Klotz, S. (2006). Urbanization and homogenization–comparing the floras of urban and rural areas in Germany. Biological conservation, 127(3), 292-30

Reviewer #2:

This study examines a question of some interest - to what degree do protected areas host unique assemblages? In general, this manuscript is well written and the assessment appears well executed. However, there are a number of areas where this study could be brought up to a higher standard and more directly address the question at hand.

**[R2.1]** Thank you for recognizing the importance of our study.

Clarifying the role of environmental uniqueness - the authors assess the role of environmental (habitat) differences between levels of protection and compositional dissimilarity (Fig 5). This is good, though the positive relationships suggest that the compositional differences between levels of protection are driven, at least partially, by the environmental differences between levels of protection. This is a key issue. Currently, it is not clear whether any differences in diversity and composition between levels of protection are due to the associated management of those systems by humans, or due to the inherent differences in the environments between those levels of protection (though acknowledged as an issue on L135-136). The first key question not currenty answered by the manuscript is: do the levels of protection differ in terms of their environmental conditions (e.g. climate, soil, oceanic attributes, etc) and how much do they differ? If the answer to this is yes, then it becomes much more difficult to make reliable inference regarding the impact of management (and protection) on those compositional attributes. This additional assessment is a crucial necessary addition to this study. If this finds fundamental environmental differences between levels of protection, then the authors need to undertake some new analyses to account for this issue if they want to draw conclusions about how protection influences diversity. I think something like a mixed effects model could be useful to partial out the effects of environmental differences and levels of protection.

**[R2.2]** The three reviewers pointed out this issue. We provided a detailed response. See **[R1.3]** We hope that improvement of our analysis fulfils the referee’s expectations. We paste our previous explanation.

We have now re-worked extensively on data and new analyses to address this important issue.

1. We provided a more complete description of environmental data and associated analyses.
2. As suggested, we used a distance-based redundancy analysis (dbRDA) to analyze changes in species composition between samples in relation to the protection level and two habitat PCA axes. Significance of the models as well as the significance of each axis and of the marginal effects were tested using ANOVA-like permutation tests. We then used a partial dbRDA, using the Jaccard turnover component, to test the effect of protection after accounting for habitat and vice-versa.
3. We also show that the habitat issue is circumvented by only considering less-strictly-protected areas within a 50-km buffer of the more protected areas (with similar results at 10 and 100km). Changes in environmental conditions should thus remain relatively limited over such spatial scales.
4. For plants, where the influence of habitat strongly explains species turnover between non-protected and protected areas, we chose a small buffer (1 km) as a reasonable range at which habitats can shape plant species occurrences. This choice is highly conservative since the probability to have different habitats between samples is higher than at larger buffer size which may cover several habitats.
5. We would like to remind that the null models we used are also a strong way to test the influence of protection on species composition while controlling for rarity, species occurrence patterns and habitat.

Altogether, we believe our results support the major role of protection in shaping species assemblages at the regional scale. Line 94

*At the scale of individual surveys, we observe a high species turnover within regions with on average 0.62 (+/- 0.14) for fish, 0.64 (+/- 0.17) for birds, and 0.97 (+/- 0.01) for plants. The distance-based redundancy analyses (dbRDA) reveal that both habitat and protection significantly explain this turnover in species composition between surveys of the same region (overall F-test = 2.8±1.9, 9.3±5.7, 35.4±9.7, mean adjusted R2 = 0.21±0.1, 0.14±0.06, 0.01±0.05, for fishes, birds and plants, respectively Fig. S1 Appendix). Yet, explanatory power of dbRDA remains low (mainly due to the sampling effect), particularly for plants, suggesting that, at the scale of surveys, many other micro-habitat variations may drive species composition beyond protection and macro-habitat effects.*

*Partial dbRDA reveal that habitat, after controlling for protection level, significantly explains species turnover between surveys of the same region for the three taxa (Fig. 2). Symmetrically, protection, after accounting for habitat, also significantly explains species turnover for the three taxa (Fig. 2). On the balance, habitat has more influence than protection on species turnover between surveys of the same region for birds (70% vs. 30%) and fish (69% vs. 31%) but this is the opposite for plants (28% vs. 56%). These results highlight that differences in habitat cannot explain, alone, the high species turnover between surveys of the same region. Since most of variation in species turnover remains unexplained between surveys within a region, owning to sampling “noise” and unmeasured micro-habitat variations, we then pool surveys by protection level for a given region with the same sampling effort in each protection level (see Methods) for the following analyses.*

2. Clarify how representative the sampled habitats are for each level of protection - another important requirement for this analysis to truely inform the effects of protection on diversity is that the sampled locations/areas are representative of those habitats and environments within each level of protection. Currently, the authors present no information on the degree to which the sampled locations reflect the different environments and habitats wihtin that level of protection.

Without such an assessment, it is impossible to say whether differences in composition and diversity are due to protection level, or due to deficiencies of data in representing all the habitats in that level of protection. Some additional analysis is required to provide the reader with more confidence that the result is not due to this type of sampling bias. In addition, some simple statistics would be useful on the total area under each level of protection, and hence the density of sample points/areas for each level of protection. Ideally this density would be relatively equivalent. Presumably the nonprotected areas have the lowest density of sample locations, so justifying the representativeness of those sample locations would be useful. Are they in the best unprotected habitat?

**[R2.3]** First, when working on large databases we have to believe that scientists in charge of surveys have likely chosen sampled locations as the most representative of habitat diversity within a given area. More importantly, during our analyses we always controlled the sampling effort. First, we determined whether a given sample was within a protected area or outside. Then, for those outside, samples within a 50km buffer of a protected area were designated as Non-protected area and retained for analyses. Because the number of sites sampled outside and inside the protected area were different, we standardized the survey effort by randomly selecting the minimum number of surveys inside and outside, 999 times. At each iteration, for each site, we took the minimum number of surveys carried out for all selected sites. Thus, the total sampled area under each level of protection was always exactly the same. In other words, our design is perfectly balanced in terms of sampled effort and area between protection levels.

We better explain this process line 123-125, 129-131, 424

Specific comments
L150-152:  "The most likely explanation for the different species compositions observed between protection levels is that species have differential responses to direct and indirect human impacts." - no, the most likely explanation is that protection levels have different environments and habitats. You need to present much stronger critical evidence if you want to make a statement like this.

**[R2.4]** see our response **[R1.3].** We appreciate the advice provided by the referee and we are now more confident in the outcomes of our study. Specifically, the partial dbRDA revealed that protection, after controlling for habitat, significantly influences species composition. We also moderated our conclusion. Line 253

*Even if our results suggest that species turnover is partly dependent on habitat, the consequence in terms of regional biodiversity conservation remains identical with Strictly Protected, Restricted and Non-Protected areas not sharing a non-negligible part of the species regional pool so being complementary. In addition, habitat influence is circumvented by only considering less-strictly-protected areas within a 50-km buffer (with similar results at 10 and 100km) around the more protected areas. In other words, change in macro-habitat (e.g. climate) remains relatively limited over our regional spatial scale. Finally, our data did not allow to determine whether a given protected area was established a prori in a specific habitat or if protection induced a differentiation of its habitat a posteriori. For instance, the management of land outside protected areas may favor certain species that are not found within more ‘natural’ habitats inside protected areas.*

L154-155: here the issue of biotic homogenisation is raised - it would be preferable to actually assess this with your data. Simply compare the levels of pairwise dissimilarity within each level of protection. This would be interesting.
**[R2.5]** The use of distance-based redundancy analysis (dbRDA) to analyze changes in assemblage composition between samples in relation to the protection level and the two habitat PCA axes allows to respond to this comment. In other words, all pairwise dissimilarities (within and between) are compared and the relative influence of both protection and habitat are tested. We unambiguously show that beyond the effect of habitat and environment, protection level significantly shapes species composition at the regional scale regardless of species detectability (rarity effect) and taxa. Line 94

Reviewer #3:

This manuscript could make a very important contribution, showing that non-protected areas can harbour substantial and unique biodiversity. This finding would be of great interest to a wide readership in biology, and has important management implications for designing protected area networks. There is a lack of papers that link protected area characteristics to biodiversity outcomes, and thus this paper could be a valuable contribution to the literature. It goes beyond existing comparisons of biodiversity inside and outside protected areas by considering turnover in species composition, which is important for stable ecosystem functioning. I have one major concern about whether the methods/results are adequate to support the conclusion that a mix of protection levels is needed to maximise the biodiversity conserved. I think this concern is probably dealt with by the methods used, but certainly needs explicit treatment in the paper.

**[R3.1]** We thank the reviewer for the very positive assessment of our manuscript and their recommendation for publication.

All of the data underlying the manuscript were already published. However, I would encourage the authors to make available a combined dataset, to make it easier to replicate their analyses. It seems the data will be released in the Github repository with the code, but I can't access the repository so I am unable to see whether this is definitely the case.

**[R3.2]** We apologize for keeping private our repository during the first round of evaluation. Our GitHub repository is now open.

I can see two interpretations for the finding that a unique set of species is found outside protected areas. The first interpretation is that the management of land outside protected areas favours certain species that are not found within the natural habitats inside protected areas. This seems perfectly plausible to me, and is the interpretation used to support the main conclusion of this paper that a mix of protection levels is required to maximise regional biodiversity. The second interpretation is that protected areas happen to be placed in a way that captures the ranges of some species but not others.

We fully agree with two plausible explanations to our patterns. Our main challenge in this revised version is to unambiguously test them.

This second aspect of the adequacy of protected areas is the topic of a large body of research (e.g. the seminal paper of Rodrigues et al., 2004, in Nature, and more recently Wintle et al., 2019, in PNAS), which is not mentioned at all in the current manuscript. The only attempt to rule out this second explanation in the current work is the demonstration that species turnover does not seem to be related to habitat diversity. However, there are many aspects of environmental turnover (climatic, habitat etc.) that might drive turnover in species composition, besides differences in land management between protected and unprotected areas. I suspect that this issue is circumvented by only considering less-strictly-protected areas within a 50-km buffer of the more protected areas. One would imagine the natural turnover of species generally to be relatively limited over such a spatial scale. Nevertheless, I would like to see some more careful testing, to ensure that the paper's main conclusion is supported by the results.

**[R3.3]** The three reviewers pointed out this issue. We provided a detailed response.

We paste our previous explanation.

We have now re-worked extensively on data and new analyses to address this important issue.

1. We provided a more complete description of environmental data and associated analyses.
2. As suggested, we used a distance-based redundancy analysis (dbRDA) to analyze changes in species composition between samples in relation to the protection level and two habitat PCA axes. Significance of the models as well as the significance of each axis and of the marginal effects were tested using ANOVA-like permutation tests. We then used a partial dbRDA, using the Jaccard turnover component, to test the effect of protection after accounting for habitat and vice-versa.
3. We also show that the habitat issue is circumvented by only considering less-strictly-protected areas within a 50-km buffer of the more protected areas (with similar results at 10 and 100km). Changes in environmental conditions should thus remain relatively limited over such spatial scales.
4. For plants, where the influence of habitat strongly explains species turnover between non-protected and protected areas, we chose a small buffer (1 km) as a reasonable range at which habitats can shape plant species occurrences. This choice is highly conservative since the probability to have different habitats between samples is higher than at larger buffer size which may cover several habitats.
5. We would like to remind that the null models we used are also a strong way to test the influence of protection on species composition while controlling for rarity, species occurrence patterns and habitat.

Altogether, we believe our results support the major role of protection in shaping species assemblages at the regional scale. Line 94

*At the scale of individual surveys, we observe a high species turnover within regions with on average 0.62 (+/- 0.14) for fish, 0.64 (+/- 0.17) for birds, and 0.97 (+/- 0.01) for plants. The distance-based redundancy analyses (dbRDA) reveal that both habitat and protection significantly explain this turnover in species composition between surveys of the same region (overall F-test = 2.8±1.9, 9.3±5.7, 35.4±9.7, mean adjusted R2 = 0.21±0.1, 0.14±0.06, 0.01±0.05, for fishes, birds and plants, respectively Fig. S1 Appendix). Yet, explanatory power of dbRDA remains low (mainly due to the sampling effect), particularly for plants, suggesting that, at the scale of surveys, many other micro-habitat variations may drive species composition beyond protection and macro-habitat effects.*

*Partial dbRDA reveal that habitat, after controlling for protection level, significantly explains species turnover between surveys of the same region for the three taxa (Fig. 2). Symmetrically, protection, after accounting for habitat, also significantly explains species turnover for the three taxa (Fig. 2). On the balance, habitat has more influence than protection on species turnover between surveys of the same region for birds (70% vs. 30%) and fish (69% vs. 31%) but this is the opposite for plants (28% vs. 56%). These results highlight that differences in habitat cannot explain, alone, the high species turnover between surveys of the same region. Since most of variation in species turnover remains unexplained between surveys within a region, owning to sampling “noise” and unmeasured micro-habitat variations, we then pool surveys by protection level for a given region with the same sampling effort in each protection level (see Methods) for the following analyses.*

See **[R1.3]** We hope that improvement of our analysis fulfils the referee’s expectations.

We also thank the referee for the reference about the adequacy of protected areas. We now discuss this point (see line 232) *A non-mutually exclusive but alternative explanation is that protected areas are placed in a way that captures the geographic range of some species but not others {Rodrigues, 2004 #116;Wintle, 2019 #432;Loiseau, 2020 #431}. Moreover, the simple occurrence within a protected area is insufficient to ensure the long-term persistence of many species, particularly those demanding particular habitat {Rodrigues, 2004 #116;Wintle, 2019 #432} or extensive home range {Dwyer, 2020 #430}. Both mechanisms may lead to the exclusion of some species from Protected areas but also increase the turnover between Protected and Non-Protected areas. In the same way, economic and social dimensions strongly influence the positioning of protected areas. For instance, the most isolated places are easier to protect due to a better social acceptance{Joppa, 2009 #437;Devillers, 2015 #438}. From this attempt to manage social-ecological systems it may result that protected species assemblages may not be representative of regional species pools, increasing species dissimilarity between protection levels. At the global scale, protection coverage tends to increase in wilderness areas to the detriment of ‘Key Biodiversity Area’ {Maxwell, 2020 #428} so regional hotspots may lack a diversity of management options including Strictly Protected areas*

My other main concern with the paper is the lack of detail given about the methods. Details of the datasets and analyses are given far too briefly to allow the study to be replicated.

We apologize for this lack of clarity.

For example:

1) Each of the datasets (and especially the datasets on French Alpine plants and on birds) are described with far too little detail.

**[R3.4]** Following this comment we provide more information about datasets

2) Similarly, there is very little information on how the protection levels were classified. Was this based on the IUCN management categories? Which categories were considered strict protected areas? What criteria were used to distinguish no-take marine reserves from other marine protected areas?

**[R3.5]** We add information on how the protection levels were classified. *“We define an area as Strictly Protected when human visitation, use and impacts are strictly controlled and limited, for instance no-take marine reserves, i.e. IUCN categories I and II (Costello 2015). In Restricted areas, human activities are controlled but some resource extraction is permitted corresponding to IUCN categories III-VI.”* See line 75

3) There needs to be more information on how the photoquadrat method was used to calculate habitat diversity for the coral reef dataset (it is not sufficient simply to refer to another paper).

**[R3.6]** #Done

4) For all of the datasets, how was the habitat information combined into a measure of habitat diversity?

**[R3.7]** #We now provide a full section entitled “Disentangle the effect of protection and habitat”. In brief habitat information was used in two different ways:

- To link beta diversity with habitat we computed Euclidean habitat distance.
- In the dbRDA we performed a PCoA on the Euclidean distance and extracted the first two axes.

5) Some more detail needs to be given on the beta-diversity
partitioning performed.

**[R3.8]** We added more details on the beta-diversity partitioning and mathematical formula.

And 6) The buffering approach needs to be explained more clearly. How was the buffering approach applied to the comparison between strict protected areas and restricted areas?
**[R3.9]** We now better explain the buffering approach and how the approach was applied to compare strictly protected areas and restricted areas:

*We collected species survey data in 655 protected areas for fish, birds and plants across the Indo-Pacific reefs, the French Alps and the US, respectively (Table S1). We define a region as a Strictly Protected or Restricted area and its surrounding Non-Protected areas within a 50km buffer, the three protection levels being present together in 45 regions. We also extracted 6, 20 and 21 habitat variables recognized as major drivers of species composition for reef fishes, birds and plants, respectively, on the same surveys (see Methods).*
Minor comments:
Title: The three levels of protection compared doesn't really constitute a 'diversity' of protection levels. Perhaps 'a mix of protection levels' would be more accurate?

**[R3.10]** We thank referee for this suggestion. We change the title accordingly but choose ‘mosaic’ instead of mix. It now read “*Maximising regional biodiversity requires a mosaic of protection levels*”.

Line 1: Perhaps change "deleterious human activities" to "anthropogenic influences"? Recognising that not all human activity is deleterious, but could still impact species diversity.

\# Done

Line 2: Perhaps avoid repetition of "different", by replacing with "distinct" protection levels.

\# Done

Lines 40-42: This last sentence could do with a rethink, as a non-protected area is not a restriction, yet on line 36 and 39 it has been noted the importance of both non-protected and protected areas.

\# Done

Line 116: It would be helpful to discuss the identity of the species unique to non-protected areas. For example, how many are invasive species?

\# We feel that this extensive series of analyses is beyond the scope of this paper

Line 204: What constitutes a "highly trained" volunteer compared to a "trained" volunteer?

#We remove highly.

Line 210: I don't see the "Habitat drivers of ß-diversity" section. Thus, the rationale for excluding certain protected areas is not clear.

\# Done

Line 242: Should this be "statuses" rather than "status".

\# Done

Line 282: What was the evidence used to select a 1-km buffer for calculating habitat diversity?

\# We chose a 1 km buffer as a reasonable range at which principal habitats of recorded species lived and to capture the relevant environmental conditions. This choice is highly conservative since the probability to have different habitats between samples is higher than at a larger buffer which may combine several habitats.

Line 289: Replace "NLCD land cover classes was…" With "NLCD land cover classes were…". Also I can only count 17 classes in the list.

\# Corrected

Lines 538 and 549: It is not clear in the figure legend what is meant by the 50-km buffer.

#Done

Figure 5: It would be helpful to have an informative label for what the y axes show, rather than just giving the protection-status comparisons.

#Done

Figure S4: The y axis labels here are not clear. The abbreviations are not defined in the figure legend.
#Done

COMMENTS FROM THE ACADEMIC EDITOR:

On a technical basis, there are some issues I’d like looking into:
* The Jaccard dissimilarity index – which is the basis of the analysis – is biased by differences in species richness (Chase et al. 2011, cited nearly 500 times now: <https://esajournals.onlinelibrary.wiley.com/doi/full/10.1890/ES10-00117.1>). It looks like the authors do employ some null modelling approach but wasn’t immediately obvious to me whether it dealt with this concern.
\# Indeed the Jaccard dissimilarity index is biased by species richness as shown by Baselga & Leprieur (2015). For this reason, we used the additive partitioning of Jaccard beta diversity that provides two separate components: (1) spatial turnover derived from species replacement which is independent of richness difference and (2) nestedness which accounts only for richness differences derived from nested patterns. In the present paper, we only discuss turnver and nestedness results.

We now added this explanation in the main text. See line 390

Baselga, A. (2010). Partitioning the turnover and nestedness components of beta diversity. Global ecology and biogeography, 19(1), 134-143. (1740 citations)

Baselga, A., & Leprieur, F. (2015). Comparing methods to separate components of beta diversity. Methods in Ecology and Evolution, 6(9), 1069-1079. (128 citations)

Baselga, A., & Orme, C. D. L. (2012). betapart: an R package for the study of beta diversity. Methods in ecology and evolution, 3(5), 808-812. (830 citations)

* In Fig. 1, it is unclear to me why the PA is a different size (i.e. circle area) in the turnover vs nestedness scenario and how much of the difference in alpha-diversity in the turnover scenario is simply because of habitat size.
\# Circles represent the size of the communities. We now better explain this in the legend.

* There should be some statistical analysis accompanying the Venn diagrams in Fig. 4. Presently there is none.

\# Venn diagrams simply represent the percentage of shared and unshared species between protection levels ( = Turnover). All corresponding statistics are provided in Figure 5.

* In Fig. 5, regression lines should are misleading and should not be drawn for non-significant relationships (all but panels B, E, H, and I).
\# Following this comment we removed regression lines for non-significant relationships.

We again thank the editors and reviewers for the considerable time and work they have dedicated to improving our manuscript.

Sincerely,

On behalf of all coauthors

Nicolas Loiseau
