# Supplementary material for: Maximizing regional biodiversity requires a mosaic of protection levels
Source: PLoS Biol. 2021 May 19;19(5):e3001195. doi: 10.1371/journal.pbio.3001195 (PMC8133472; doi:10.1371/journal.pbio.3001195)
Supplement: Attachment — Submitted filename: responsetoreviewers_R2.docx [file pbio.3001195.s010.docx]

Dear Dr Roberts,

We would like to thank you for handling our manuscript during several rounds of reviews. We are also grateful to the Academic Editor and the reviewers for the constructive comments which significantly improved our manuscript. We are pleased to submit the final version of our manuscript entitled *Maximising regional biodiversity requires a mosaic of protection levels* for publication in *Plos Biology*.

We feel that our title is informative and accessible to a large readership. We now explicitly include “gamma” or regional diversity in our estimates as suggested by reviewers #1. In fact, we felt that the link between beta and gamma diversity was implicit in our previous version. We now mention gamma diversity in our Figure 1 and in the introduction. We also extent the discussion on this point with results supported by Figure 7.

However, we hope that keeping the same title is not an obstacle for publication. As an alternative, we could suggest the following title: *Adjacent distinct protection levels host different species compositions.*

We believe that our MS has greatly benefitted from these changes. Point-by-point responses to all comments are provided below, and we enclose a revised version of the manuscript with all changes highlighted (line numbers refer to the revised manuscript with track-changes). We can address any remaining concerns

Thank you for your time and trouble.

Sincerely,

On behalf of all coauthors

Dr. Loiseau

Dear Dr Loiseau,

Thank you for submitting your revised Research Article entitled "Maximising regional biodiversity requires a mosaic of protection levels" for publication in PLOS Biology. I have now obtained advice from two of the original reviewers and have discussed their comments with the Academic Editor. 

Based on the reviews, we will probably accept this manuscript for publication, provided you satisfactorily address the remaining points raised by the reviewers. Please also make sure to address the following data and other policy-related requests.

IMPORTANT:

a) Please address the remaining concerns raised by reviewers #1 and #3.
#We responded to all comments below.

b) Note that reviewer #1 takes issue with your current title. However you address this concern, please ensure that the revised title is informative and accessible to our broad readership.

\# We chose to explicitly include gamma or regional species diversity in this new version so the title can remain the same since informative, accessible and attractive for a broad readership.

c) Please also address the points mentioned by the Academic Editor at the foot of the email. Unfortunately reviewer#2 was unable to re-review, so I'd asked the Academic Editor to assess your responses to rev #2's concerns, in addition to those to his/her own comments.
#We thank the Academic Editor for his/her comments and responded below.

d) Please attend to my Data Policy requests further down, namely to supply the numerical values underlying Figs 2ABC, 3ABC, 4ABCDEFGHI, 5ABCDEFGHI, 6ABCDEFGHI, S1, S2, S3, S4, S5, and to cite the location of these data in all respective Figure legends.
#We dropped all necessary data to reproduce figures to a publicly available repository (https://github.com/LoiseauN/Betadiversity-protected-areas) and provide location of these data in all respective Figure legends.

As you address these items, please take this last chance to review your reference list to ensure that it is complete and correct. If you have cited papers that have been retracted, please include the rationale for doing so in the manuscript text, or remove these references and replace them with relevant current references. Any changes to the reference list should be mentioned in the cover letter that accompanies your revised manuscript.
#We review our reference list. Following comments, we added several references:

Updated references on changing diversity: Daskalova et al. 2020, Rishworth et al. 2020, Su et al. 2021, Yan et al. 2021

Recent references on effectiveness of protected areas : Claudet, et al. 2021

Reference ask by reviewer #3 : Dudley 2008

Updated reference on biodiversity trends through time Rishworth, Adams et al. 2020

Updated Reference on expansion of protection to environmental conditions Hanson et al. 2020, Rishworth, et al. 2020

Claudet, J., C. Loiseau and A. Pebayle (2021). "Critical gaps in the protection of the second largest exclusive economic zone in the world." Marine Policy 124: 104379.

Daskalova, G. N., I. H. Myers-Smith, A. D. Bjorkman, S. A. Blowes, S. R. Supp, A. E. Magurran and M. Dornelas (2020). "Landscape-scale forest loss as a catalyst of population and biodiversity change." Science 368(6497): 1341-1347.

Dudley, N. (2008). Guidelines for applying protected area management categories, Iucn.

Hanson, J. O., J. R. Rhodes, S. H. Butchart, G. M. Buchanan, C. Rondinini, G. F. Ficetola and R. A. Fuller (2020). "Global conservation of species’ niches." Nature 580(7802): 232-234.

Rishworth, G. M., J. B. Adams, M. S. Bird, N. K. Carrasco, A. Dänhardt, J. Dannheim, D. A. Lemley, P. A. Pistorius, G. Scheiffarth and H. Hillebrand (2020). "Cross-continental analysis of coastal biodiversity change." Philosophical Transactions of the Royal Society B 375(1814): 20190452.

Su, G., M. Logez, J. Xu, S. Tao, S. Villéger and S. Brosse (2021). "Human impacts on global freshwater fish biodiversity." Science 371(6531): 835-838.

Yan, H. F., P. M. Kyne, R. W. Jabado, R. H. Leeney, L. N. Davidson, D. H. Derrick, B. Finucci, R. P. Freckleton, S. V. Fordham and N. K. Dulvy (2021). "Overfishing and habitat loss drive range contraction of iconic marine fishes to near extinction." Science Advances 7(7): eabb6026.

We expect to receive your revised manuscript within two weeks.

To submit your revision, please go to <https://www.editorialmanager.com/pbiology/> and log in as an Author. Click the link labelled 'Submissions Needing Revision' to find your submission record. Your revised submission must include the following:
-  a cover letter that should detail your responses to any editorial requests, if applicable, and whether changes have been made to the reference list
-  a Response to Reviewers file that provides a detailed response to the reviewers' comments (if applicable)
-  a track-changes file indicating any changes that you have made to the manuscript. 

NOTE: If Supporting Information files are included with your article, note that these are not copyedited and will be published as they are submitted. Please ensure that these files are legible and of high quality (at least 300 dpi) in an easily accessible file format. For this reason, please be aware that any references listed in an SI file will not be indexed. For more information, see our Supporting Information guidelines:
<https://journals.plos.org/plosbiology/s/supporting-information>  

*Published Peer Review History*
Please note that you may have the opportunity to make the peer review history publicly available. The record will include editor decision letters (with reviews) and your responses to reviewer comments. If eligible, we will contact you to opt in or out. Please see here for more details:
<https://blogs.plos.org/plos/2019/05/plos-journals-now-open-for-published-peer-review/>

*Early Version*
Please note that an uncorrected proof of your manuscript will be published online ahead of the final version, unless you opted out when submitting your manuscript. If, for any reason, you do not want an earlier version of your manuscript published online, uncheck the box. Should you, your institution's press office or the journal office choose to press release your paper, you will automatically be opted out of early publication. We ask that you notify us as soon as possible if you or your institution is planning to press release the article.

*Protocols deposition*
To enhance the reproducibility of your results, we recommend that if applicable you deposit your laboratory protocols in [protocols.io](http://protocols.io/), where a protocol can be assigned its own identifier (DOI) such that it can be cited independently in the future. For instructions see: <https://journals.plos.org/plosbiology/s/submission-guidelines#loc-materials-and-methods> 

Please do not hesitate to contact me should you have any questions.

Sincerely,

Roli Roberts

Roland G Roberts, PhD,
Senior Editor,
[rroberts@plos.org](mailto:rroberts@plos.org),
PLOS Biology


------------------------------------------------------------------------
DATA POLICY:

You may be aware of the PLOS Data Policy, which requires that all data be made available without restriction: <http://journals.plos.org/plosbiology/s/data-availability>. For more information, please also see this editorial: <http://dx.doi.org/10.1371/journal.pbio.1001797> 

Note that we do not require all raw data. Rather, we ask that all individual quantitative observations that underlie the data summarized in the figures and results of your paper be made available in one of the following forms:

1) Supplementary files (e.g., excel). Please ensure that all data files are uploaded as 'Supporting Information' and are invariably referred to (in the manuscript, figure legends, and the Description field when uploading your files) using the following format verbatim: S1 Data, S2 Data, etc. Multiple panels of a single or even several figures can be included as multiple sheets in one excel file that is saved using exactly the following convention: S1_Data.xlsx (using an underscore).

2) Deposition in a publicly available repository. Please also provide the accession code or a reviewer link so that we may view your data before publication. 
#We dropped all necessary data to reproduce figures to a publicly available repository (https://github.com/LoiseauN/Betadiversity-protected-areas).

Regardless of the method selected, please ensure that you provide the individual numerical values that underlie the summary data displayed in the following figure panels as they are essential for readers to assess your analysis and to reproduce it: Figs 2ABC, 3ABC, 4ABCDEFGHI, 5ABCDEFGHI, 6ABCDEFGHI, S1, S2, S3, S4, S5. NOTE: the numerical data provided should include all replicates AND the way in which the plotted mean and errors were derived (it should not present only the mean/average values).
#All data to reproduce figure are in the folder outputs from our github.

IMPORTANT: Please also ensure that figure legends in your manuscript include information on where the underlying data can be found, and ensure your supplemental data file/s has a legend.
We provide location of data to draw figures in all respective Figure legends

Please ensure that your Data Statement in the submission system accurately describes where your data can be found.

------------------------------------------------------------------------
DATA NOT SHOWN?

- Please note that per journal policy, we do not allow the mention of "data not sown", "personal communication", "manuscript in preparation" or other references to data that is not publicly available or contained within this manuscript. Please either remove mention of these data or add figures presenting the results and the data underlying the figure(s).
------------------------------------------------------------------------

REVIEWERS' COMMENTS:


Reviewer #1:

I was reviewer 1 in the previous round. I'm largely satisfied with the responses of the reviewers to my concerns, especially regarding beta-diversity calculations. I'm a bit less certain why the authors have chosen to ignore (i.e., no measure/report) some indication of larger scale (gamma) diversity. But I will not quibble too much. My remaining concern, then, lies in the title.  The authors are discussing 'maximizing' regional biodiversity, but not directly measuring it. I understand that the Baselga measures get you part way there, but why not just measure regional diversity itself?  Otherwise, the title seems misleading, as well as bits of the abstract. Scale is a real thing in biodiversity studies, and beta diversity, at least as measured here, obscures it as much as helps it, whereas measuring richness and other measures at multiple scales (ie., SARs, rarefaction curves or the like) allow you to directly look at this.
\# We thank the reviewer for the positive assessment of our manuscript, and his recommendation for publication. We appreciate the reviewer effort that greatly improved our manuscript during this long process.

Gamma diversity is an important component that we now carefully and explicitly address. We agree that gamma diversity can be simply measured and added to our hypotheses.

First, we mention gamma diversity in our new figure 1 and in our introduction along with hypotheses. Second, Venn diagrams in Figure 7 can be interpreted as the percentage of gamma diversity. We extended the discussion to provide a more straightforward message on gamma/regional diversity.

See line 89-90; 280-281, 352-355

*“As an alternative, protected and non-protected may host different species inducing a mechanical increase in γ-diversity (Fig. 1).”*

*“Together, Restricted and Strictly Protected areas host, on average, 88, 87 and 85% of bird, fish and plant species regional diversity, respectively.”*

*“For birds and plants, our results also highlight the critical role of conservation efforts in the protection of imperilled species, since together Restricted and Strictly Protected areas host 94 and 62% of the regional and imperiled species diversity, respectively.”*

Then, we feel that our title is in line with our content since species turnover among areas mechanically increases the level of regional diversity. We keep our title which is informative and accessible to a large readership. It highlights the main result of our paper: a reinforcing Strictly Protected areas with Restricted areas nearby may create a mosaic of protection levels maximizing the number of species at the regional scale

Reviewer #3:

the authors have done a very thorough job of responding to the comments made by myself and the other reviewers. In particular, they have added extra analyses to parse out the contributions of habitat versus protection differences on observed turnover in species composition. I have a few suggestions for changes to the text that I hope may further improve the paper.

We thank the reviewer for the positive assessment and appreciate her/his effort that greatly improved our manuscript.

My main remaining concern is around the conservation recommendations that emerge from the findings. On lines 253-256, the authors contend that consequences are identical whether or not habitat plays a substantial role in driving observed differences in species composition. I don't agree. If it is the climate component of habitat differences that drive the differences in species composition, then it does not necessarily follow that having a mosaic of different protection levels will benefit biodiversity. Expanding protection to the climates (and thus species) that are not already protected would be most beneficial. Along the same lines, the authors respond that considering the identity of the species found in different protection levels (including whether or not they are invasive species) is beyond the scope of the paper. That is perfectly reasonable, but it would be good to add some discussion of the conservation implications of species identity. Different conservation perspectives will place different values on different species, which is important for informing management recommendations.

\# We now discuss the extension of protection to climates and the identity of species in the main text. See line 451-453, 457: 461

*However, from a conservation perspective, expanding protection to environmental conditions that are not already or weakly protected would be highly beneficial for imperiled species (Hanson, Rhodes et al. 2020).*

*Finally, future investigation on species identities and functions would reveal mechanisms underlying the high species turnover we observe. Can disparity in trophic level or other functional traits explain changes in species composition between protection levels? What is the contribution of non-native species to the turnover between these management levels?*

Related to my previous comment, I am not sure about referring to habitat dissimilarity, since the measures of 'habitat' include climate as well as land cover, reef structure etc. The reader has to get a long way through the paper (until the Methods section at the end) before realising that the methods controlled for climatic differences. I wonder if a more inclusive label could be used? Or at least, it would be good to state early on that climate was included in the analysis.
#Agree, we early define “environmental conditions” that include both habitat and climatic data.

See lines 75-76

The supplementary analysis of the standardised effect sizes highlights that strictly protected areas are important for sustaining reef fish biodiversity. But I don't think this important result is discussed in the main text.
#Agree, we previously discussed that point in the supporting information. We added a sentence about the importance of strict protection to sustain reef fish biodiversity. See lines 240-241

Specific comments:
Lines 65-68: This sentence doesn't follow neatly from the previous one.

We replace So, on the balance by thus see line 77
Lines 80-81: It is odd to talk of a 'pure' conservation effect. In reality, outcomes will depend on a complex mix of abiotic factors, natural biotic processes, and human activities.

#We removed “pure”
Line 83: This would be a good point at which to state that the 'habitat' effects also included measures of climate.

#Done
Lines 101-102: The turnover numbers presented here won't be intuitive for readers unfamiliar with the specific indices used. Could the statistics be restated in a way that is easier to interpret?

#Done, turnover can be interpreted as a percentage (line 137)
Line 107: The 'sampling effect' hasn't been introduced yet.

#Done see line 139, 145-146
Line 119: "owing to"

#Done
Lines 119-121: The reason for pooling surveys within protection levels needs unpacking in more detail here.

#Done see line 146
Line 126: "as in" not "than in"

#Done
Line 132: In what sense is this "symmetrically"?

#We removed symmetrically
Line 132: Clarify whether these "habitat differences" also include the climate variables.

#Now environmental differences
Line 141-143: The rationale for the null modelling approach introduced here needs more explanation.

#Done see line 236-237
Line 148-150: Do the statistics presented in this sentence reflect the results for all datasets combined?

#Yes, we better explained. See line 243
Line 155: Should this be "strongly nested within plant diversity"?

#Done
Line 162: Again, clarify whether the "habitat difference" here included the effects of the climate variables.

#Now environmental differences
Lines 168-169: For birds at least, Figure 6 seems to show a significant positive relationship for all three comparisons.

#Done
Line 197: It hasn't been very clear yet how the methods accounted for the effects of species rarity when rare species (species with a number of occurrences lower than 3 within a region) are removed (Fig. S4 Appendix).

#More details, see line 363
Line 243: "Key Biodiversity Areas"

#Done
Line 259-260: I am not convinced by the argument that "change in macro-habitat (e.g. climate) remains relatively limited over our regional spatial scale". In many regions, climate can change markedly over 50 km. For example, in areas of high topographic complexity (such as the Alps!).

#Agree we nuanced our sentence. However, it is important to note that at 10km, where climate differences are limited, we found similar results.
Line 302: "The database included" not "Database include"

#Done
Lines 312-313: For the analysis of the bird dataset, presumably the BBS routes had to fall entirely within a given protection level to be included in the analysis?

\# If the middle of the road was in a given protection, it was included in the analyses
Line 319-323: These estimates of 8-13% species considered imperiled are much lower than the %s reported in the IPBES global assessment. Is that just to do with the regions considered?

\# Indeed, this difference could be due to fact that we did not work at global scale. For instance, for birds and plants, US and French alps are not the strongest impacted areas.
Line 330: Give a reference where the criteria for distinguishing IUCN categories are given in full.

#Done
Lines 330-331: Specify here again that the 'Restricted Areas' correspond with IUCN categories III-VI.

#Done
Lines 333-334: Say a little more about the origin of these "local data".

#Done
Line 368: Is 'average precipitation' the average of total annual precipitation, and 'temperature' mean annual temperature?

#More precision added
Lines 369-370: What choice is highly conservative? In what sense is it highly conservative?

\# We removed this sentence that was unclear
Line 373: 'US land cover' is not an informative title, given that both land cover and climate data were included.

#Done
Lines 383-384: How was the 1 km spatial resolution of the climate data reconciled with the fact that the BBS routes spanned 40 km?

\# We averaged the climatic data within the 19.7km-radius circle. See line 671
Lines 410-414: Was it possible to have a comparison consisting of just restricted areas and non-protected areas? Was the same 50-km buffering approach applied?

\# The same 50 km buffering approach was applied on restricted areas allowing comparison between restricted and non-protected areas. See line 776
Lines 419-420: The reason for this pooling is never made very clear.

#Pooling surveys allows to limit the sampling ‘noise’. See lines 712, 139-140
Lines 422-423: Wasn't the buffering approach intended to control, as much as possible, the climatic conditions surveyed? Potential spillover of species is a separate issue.

#Done
Line 452: "applied a similar approach as taxonomic beta diversity".

#Done
Line 454: Specify here whether the "habitat information" included the climate data.

#Done
Lines 466-467: Was the PCoA based on the habitat information? Did this include the climate data?

#PCoA also includes climate data, see line 768
Line 469: Under what circumstances were the PCoA axes not available?

#We corrected the sentence which now reads: “*Each survey was associated with its protection level and when environmental conditions were available, 2 PCoA axes as explanatory variables.* "
Lines 472-474: More details are needed here on the computation of the partial dbRDA.

#Done
Lines 676-677: I.e. whether or not each of habitat and protection had a significant association with observed turnover?

#Done
Lines 699-700: "a random allocation of species to surveys"

#Done
Figure 5: It is odd to use "turnover" as the y-axis label, when turnover is just one of the components of beta diversity considered. Perhaps use "dissimilarity".

#In this figure, we plot the turnover and not the overall beta diversity or dissimilarity.
Supplementary Information, Figure S3 legend: Does "at 100km and 10km" refer to the buffers of 100 km and 10 km?

#Yes we corrected that point
Figure S5 legend: "significance" not "significativity"

#Done


COMMENTS FROM THE ACADEMIC EDITOR [lightly edited]

The response to my comments seem fine, but the addition of "circle area" to the Fig. 1 caption doesn't fit for the Nestedness case, as the circle with three black birds on the far left (Nestedness = 1) is smaller than the other two circles that also have 3 species. Seems trivial but for these types of schematics to work, they have to be very clear.

#Agree, we corrected that point.

As for reviewer #2's comments, I agree the major ones mostly overlap with the other Reviewers, who seem satisfied. The one exception is the comment about L154-155 in the original MS with response R2.5 from the authors. I don't understand what the authors have done, if anything, to address this comment.

This point was addressed with the distance-based redundancy analysis (dbRDA). In fact, for this approach, all pairwise dissimilarities (within and between) are compared.
